# Efficacy of peroneal nerve functional electrical stimulation (FES) for the reduction of bradykinesia in Parkinson’s disease: an assessor-blinded randomised controlled trial (STEPS II)—study protocol

**DOI:** 10.1136/bmjopen-2024-097010

**Published:** 2025-09-05

**Authors:** Abbey Tufft, Helen Neilens, Jonathan Marsden, Siobhan Creanor, Ayesha Ali, Margaret Donovan-Hall, Paigan Aspinall, Amber Lord, Ben Jones, Paul Taylor

**Affiliations:** 1 Peninsula Clinical Trials Unit, Faculty of Health, University of Plymouth, Plymouth, UK; 2 Faculty of Health, School of Health Professions, University of Plymouth, Plymouth, UK; 3 Exeter Clinical Trials Unit, Faculty of Health and Life Sciences, Department of Health and Community Sciences, Exeter Medical School, University of Exeter, Exeter, UK; 4 School of Health Sciences, University of Southampton, Southampton, UK; 5 Salisbury NHS Foundation Trust, Salisbury, UK; 6 Odstock Medical Limited, Salisbury, UK

**Keywords:** Gait, Wearable Devices, Parkinson-s disease, Electric Stimulation Therapy

## Abstract

**Introduction:**

Difficulty with walking can lead to reduced quality of life for people with Parkinson’s disease (pwPD); improving walking is considered a treatment priority. Drug therapies can control PD symptoms; however, pwPD often still experience mobility problems.

Functional electrical stimulation (FES) induces movement in weak muscles via external electrical stimulation. FES is used in stroke and multiple sclerosis patients to correct dropped foot by stimulating the common peroneal nerve and is associated with improved quality of life and mobility. The randomised feasibility study preceding this definitive study showed that daily FES can produce a clinically meaningful improvement in walking speed in pwPD; this was sustained 4 weeks after FES was withdrawn. STEPS II is the first definitive randomised controlled trial, with blinded outcome assessment, aiming to determine the efficacy of FES in pwPD.

**Methods and analysis:**

STEPS II is a two-group, parallel, assessor-blinded, superiority randomised controlled trial with an internal pilot, designed to compare FES plus usual care versus usual care alone. 234 participants will be randomised across eight UK sites. Telephone pre-screening and face-to-face screening will determine eligibility. The intervention group will attend four unblinded FES visits to receive the device and assess walking with and without FES. All participants have blinded assessments at baseline and weeks 2, 6, 18 and 22. The primary objective is to compare whole body bradykinesia at 18 weeks post-baseline via changes in 10m walking speed. Secondary objectives will assess the wider effects of FES on Parkinsonian gait and quality of life. An embedded qualitative component will explore wider experiences of FES.

**Ethics and dissemination:**

This study received ethical approval from the Yorkshire and The Humber-Sheffield Research Ethics Committee (reference 23/YH/0193). A Data Monitoring Committee and Trial Steering Committee will provide independent oversight. Dissemination will be via publications, conferences and social media. FES intervention and training materials will be made open access.

**Trial registration number:**

ISRCTN13120555.

**STRENGTHS AND LIMITATIONS OF THIS STUDY**

- This is the largest study investigating the effects of functional electrical stimulation (FES) in people with Parkinson’s disease (pwPD). The sample size has sufficient statistical power to determine if FES is an efficacious treatment for pwPD.
- This is an assessor-blinded study; it was not possible to blind the participants as there is no credible sham placebo device.
- The study has ecological validity as it uses standard FES provisions over a realistic intervention period.
- This study will provide an indication of underlying mechanisms of action that can inform future studies.

## Introduction

### Background and rationale

 Parkinson’s disease (PD) affects about 148 000 people in the UK and is growing in incidence faster than any other neurological condition.^1 2^ Difficulty in walking is a major factor reducing quality of life for people with Parkinson’s disease (pwPD) and is considered a priority for treatment.^3^ Walking is often unsafe, and pwPD are recurrent fallers.^4 5^ These issues can lead to a reduction in overall activity, fitness, mobility, health status and participation in society; it is common for pwPD to become socially withdrawn.^6^

Parkinsonian gait is characterised by bradykinesia (slow movement), hypokinesia (small movement), festination (rapid short strides), akinesia (difficulty initiating movement) and freezing (sudden and temporary episodes of inability to move forward). Walking is often asymmetric, with deficits in interlimb coordination and reduced muscle activity, particularly affecting distal muscles and the ability to dorsiflex (lift) the feet.^7 8^ These deficits are associated with slow walking and freezing.^9^

The symptoms of PD can be controlled by drug therapies that modify dopamine action in the brain.^10^ However, even with medication, pwPD still experience mobility and balance problems. Further, medication is limited in its effectiveness over the course of the day and as the disease progresses.^11^ PD medication also has side effects, which may lead to poor adherence. Exercise-based therapies are often used to supplement the effects of PD medication.^12 13^ More recently introduced interventions include deep brain stimulation and transcranial stimulation^14^; however, these procedures are invasive, high risk and costly.^13^ Despite available interventions, difficulty in walking is still a major contributor to reduced quality of life, and research into improving walking ability and reducing falls remains a priority for pwPD.^3 5 15^

Functional electrical stimulation (FES) uses externally applied electrical stimulation to induce functional movement in paralysed or weak muscles. It is commonly used and recommended in England by the National Institute for Health and Care Excellence for correcting dropped foot in stroke and multiple sclerosis (MS) patients by stimulating the common peroneal nerve, in time with the swing phase of gait, causing dorsiflexion.^16^ The benefit is demonstrated by increased walking speed while using the device^17^,^19^; this is a good indicator of gait quality and correlates with the level of functional walking.^20^ FES use in stroke and MS is also associated with a reduction in the incidence of falls,^21 22^ reduced effort of walking^17 23^ and improved quality of life.^24 25^ Further initial studies have suggested that FES can reduce freezing in pwPD.^26 27^

The difference in walking speed with and without FES, measured on the same occasion, is referred to as an ‘orthotic effect’.^14^ FES can also have a ‘therapeutic effect’, where an increase in walking speed is observed without FES assistance, after a period without using the device. A short-term therapeutic effect is referred to as a ‘carryover effect’ and may relate to increased excitability of the neurological system and short-term adaptive changes.^28^ If the therapeutic effect is maintained for longer, it is referred to as a ‘training effect’ and may be due to additional mechanisms such as muscle strengthening and motor relearning.^29^ Typically, the orthotic effect is reported to be more significant than the therapeutic effect in people with MS or stroke.^17 30^

In the STEPS feasibility study (The Effectiveness of Peroneal Nerve Functional Electrical STimulation (FES) for the Reduction of Bradykinesia in Parkinson’s: A Pragmatic Feasibility Study for a Single Blinded Randomised Control Trial: STEPS), 64 pwPD were recruited and randomly allocated to receive either usual care or FES with usual care for 18 weeks, followed by 4 weeks of FES withdrawal.^31^ The mean total orthotic effect in pwPD was similar to that observed in stroke and MS patients. However, the mean therapeutic effect observed was greater than that observed in stroke and MS patients. An improved mean walking speed in favour of the intervention group was observed after 6 weeks, maintained at 18 weeks and still present at 22 weeks (4 weeks after FES was withdrawn).^31^ Other research also indicates that the effects of FES in pwPD occur after only a short period of FES use^32 33^ and that the effects of FES in pwPD occur after only a short period of FES use and that it can improve symptoms of freezing.^26 27^

This initial observation in pwPD suggests there is a different treatment effect to that observed in FES users with other neurological conditions. Therefore, further research is needed to determine the potential mechanisms of action in pwPD.

Mechanisms of action will be explored by assessing short-term changes in interlimb coordination, dynamic balance and limb bradykinesia and assessing their relative contributions in improving walking speed after 6 and 18 weeks. Changes in weakness in muscles directly targeted and not targeted by the intervention will be characterised over time and the impact of sensory dysfunction will also be investigated.

Database searches indicate there are no other registered randomised controlled trial (RCT) studies, with blinded outcome measures, assessing the effect of FES in pwPD. This study fills a gap in the literature and addresses the need for high-quality trials exploring the effect of FES in pwPD.^34^

### Objectives

Primary objective: to compare whole body bradykinesia of pwPD at 18 weeks post-baseline using FES with usual care versus usual care alone.

Secondary objectives:

- To determine if any effects remain 4 weeks after FES is withdrawn.
- To investigate the effect of FES on other aspects of Parkinsonian gait and living life with PD, to inform the need and design of future research. To achieve this, we will produce estimates of effect size of FES for:
  - Hypokinesia.
  - Akinesia.
  - Falls and balance.
  - Activities of daily living.
  - Activity.
  - Quality of life.
  - Cost/utility.
  - The effect on gait while using FES.
- To determine how pwPD and their carers perceive the usefulness and practical experience of FES use and its therapeutic effect through an embedded qualitative component.
- To determine the safety of FES in pwPD.
- To investigate potential mechanisms of action of FES in PD by determining:
  - Short-term (up to 6 weeks) changes in interlimb coordination, anticipatory postural adjustments (APA) and limb bradykinesia while stepping and walking.
  - The link between putative mechanisms of action and their relationship to walking speed at 6 and 18 weeks through causal mediation analysis.
  - Changes over time in the strength of muscles directly targeted and not targeted by the intervention and the impact of sensory dysfunction.

## Method and analysis

### Patient and public involvement

The STEPS II Patient Advisory Group is composed of pwPD and family members/carers, and meets two times per year. They are involved at all stages of the study. They supported discussions around suitability of outcome measures, design and review of participant-facing documentation and methods to improve recruitment and retention. They will be involved in producing lay results and advising on suitable methods to disseminate this information.

### Trial design and setting

STEPS II is a multicentre, two-group, parallel, assessor-blinded, superiority, individual RCT with an internal pilot phase, comparing FES plus usual care versus usual care alone.

The study is set at secondary care National Health Service (NHS) Trusts in England and Wales, with eight recruiting sites. Potential sites were assessed for feasibility by presence of an established FES service and suitable staff to assume the blinded assessor role who had experience assessing walking in pwPD.

Potential participants are identified at their routine neurology or physiotherapy outpatient appointments, where they are introduced to the study and provided with a recruitment pack. They are asked if they wish to receive a follow-up pre-screening telephone call from a member of the study team. If they do not wish to receive a call, they can use the contact details in the recruitment pack to contact the study team. Patient databases may also be reviewed and the recruitment pack posted to potential participants.

### Eligibility criteria

Patients must satisfy the following inclusion criteria:

- Aged 18 years and above (no upper age limit).
- Idiopathic PD.
- Hoehn and Yahr stages I–IV.
- Difficulty in walking due to PD bradykinesia, defined as a measured 10 metre walking speed (10mWS) of less than 1.25 m/s. The maximum 10mWS for inclusion in the study was raised from 0.8m/s to 1.25 m/s 5 months into the feasibility study because many pwPD who were initially screened were rejected at assessment, despite reduced ankle movements, because they walked too fast. Increasing the threshold for inclusion to 1.25 m/s better reflects the cohort of pwPD who have walking problems. This speed also has practical meaning for community mobility as it is the minimum speed needed to safely use a pelican crossing.^35^
- Able to walk 50 m with appropriate walking aids, but without assistance from another person. Appropriate aids include walking sticks, tri-sticks or quad-sticks, ankle foot orthoses and similar devices.
- Able to achieve standing from sitting without the assistance of another person.
- Able to understand and comply with the treatment and assessment procedures.
- Able to give written informed consent

Patients who meet any of the following exclusion criteria will be ineligible:

- Receiving, or scheduled to start receiving, deep brain stimulation within the next 6 months.
- Scheduled to start apomorphine, Duodopa or Produodopa within the next 6 months (those who are currently taking Duodopa and apomorphine are eligible).
- Pyramidal and/or extrapyramidal system injuries.
- Untreated or refractory epilepsy with seizures in the last 3 months.
- Pregnancy or planned pregnancy.
- Cardiac pacemaker or other active medical implanted devices.
- Denervation of the common peroneal nerve, or other neurological condition known to cause dropped foot.
- Severe osteoarticular pathology that involves the calf bones, knee and tibiotarsal joints, or other conditions that significantly affect walking.
- Malignancy or dermatological conditions in the leg that would be stimulated.
- Major cognitive impairment (eg, dementia).
- Under treatment for an unresolved deep vein thrombosis in the leg that would be stimulated.
- Participating in another interventional clinical trial.

### Intervention and comparator

Participants allocated to the intervention group will receive the FES intervention alongside their usual care.

**Figure 1:**
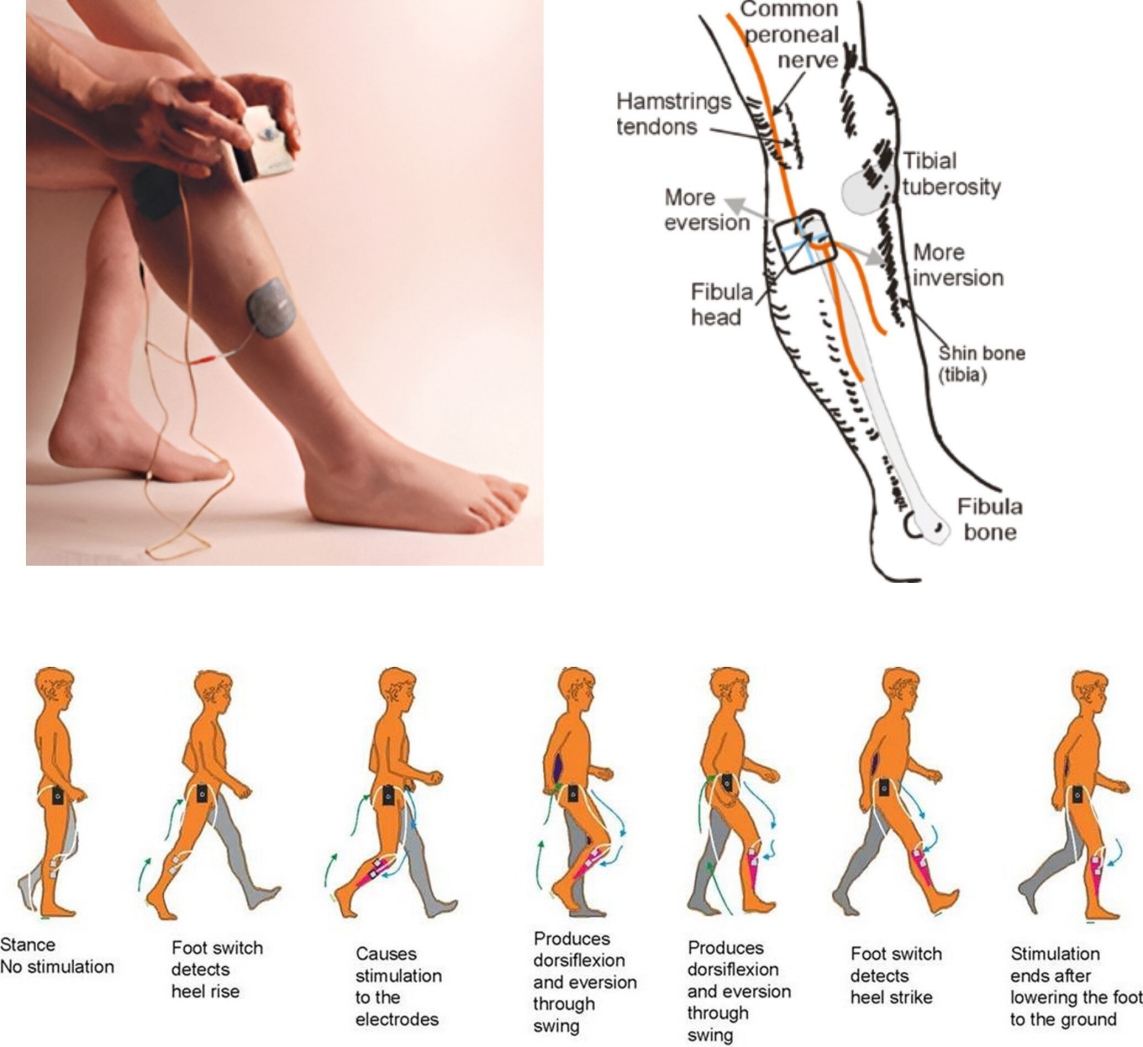
The ODFS Pace with electrode positions shown relative to the underlying common peroneal nerve. ODFS, Odstock Dropped Foot Stimulator.

The European Conformity marked (CE-marked) Odstock Dropped Foot Stimulator (ODFS) Pace is a small, battery-powered, single-channel FES device used to correct dropped foot. Electrical stimulation is applied to the common peroneal nerve using skin surface electrodes placed over the head of fibula and the anterior tibialis muscle. Stimulation is timed to the individual’s gait cycle using a footswitch in the shoe, causing dorsiflexion when the foot is raised (see figure 1).

Intervention participants will attend four appointments with an unblinded FES clinician at weeks 1, 2, 6 and 18 post-baseline. This is the FES clinical pathway established for stroke and MS patients.^36^

During the first FES set-up appointment, the clinician will fit the device to the leg that has the greatest deficit in ankle movement. Stimulation intensity will be set to cause a comfortable contraction, producing dorsiflexion and eversion. The participant will be taught how to fit the device, identify the correct movement and adjust stimulation intensity. Two 10 m walking tests (10mWT) and a Timed Up and Go (TUG) test will be carried out prior to the first use of the device. Intervention participants are asked to walk every day with the device and have a contact number for the local clinician to report problems or device-related adverse events.

Participants will attend a second set-up appointment during week 2; the clinician will provide additional training, if required. Two FES follow-up visits take place during weeks 6 and 18. During the second set-up and follow-up visits, participants will carry out 10mWTs (with and without FES) and TUG tests (with and without FES), and will report Borg Rating of Perceived Effort scores. The clinician will record consumables used and contact time; this data will be used to determine cost/utility of intervention delivery. Intervention participants will complete two questionnaires (FES experience questionnaire and Systems Usability Scale) at week 18 to explore their experience with FES.

At each FES appointment, the clinician will extract data from the ODFS Pace logger, including the number of steps taken while using the device, total stimulation time, intensity and pulse width, to determine the frequency of device use and adherence in between visits.

Participants in the control group will receive their usual care. Usual care includes PD medication (dose/frequency) and participation in physiotherapy or exercise (type of exercise/duration). Data on usual care will be collected for all participants; medication will be recorded at each blinded assessment and participation in exercise and physiotherapy will be recorded by the participant in weekly exercise diaries.

### Outcome measures

The primary outcome measure is the 10mWS at the week 18 blinded assessment.

The following outcome measures are secondary and will be recorded for all participants at each blinded assessment visit:

- 10mWS.
- Stride length during the 10mWT.
- Movement Disorder Society-Unified Parkinson’s Disease Rating Scale (MDS-UPDRS) sections 1a, 1b, 2 and 3.^37^
- ‘New’ Freezing of Gait Questionnaire.^38^
- Falls Efficacy Scale International Questionnaire.^39^
- Mini-Balance Evaluation Systems Test.^40^
- Parkinson’s Disease Questionnaire-39.^41^
- European Quality of Life 5 Dimension 5 Level (EQ-5D-5L) Questionnaire.^42^
- Falls and exercise diary.

Mechanistic outcome measures will be recorded during the baseline, week 2 and week 6 blinded assessments. This data will be collected via Physilog (Switzerland) inertial measurement units (IMUs) during the 10mWT, MDS-UPDRS toe tapping task and APA stepping task. Muscle strength will be measured via handheld ActivForce dynamometry during dorsiflexion, hip flexion and eversion. Sensory thresholds will be assessed at baseline using a 2-point discrimination test and a gradient sensory discrimination test.^43^ ^44^ IMU^45^ data will be used to determine:

- Phase Coordination Index during the 10mWT.^46^
- Changes to APA during the stepping task.^47^
- Changes in limb bradykinesia during the toe tapping task and 10mWT.

Daily step count will be recorded via StepWatch pedometers (Modus, USA) worn 7 days prior to baseline, week 18 and week 22 blinded assessments.

### Data collection methods

Blinded assessments involve data collection via patient-reported outcome measures (PROMs) and physical assessments. PROMs may be completed online or on paper, depending on the participant’s preference. Blinded assessors, typically a physiotherapist or clinical scientist, who have been trained in the outcome measures will assess outcomes and collect the data. They will enter the data either directly into the study database or on paper case report forms to be entered into the database retrospectively. Assessors will complete a sense check on PROMs to ensure they have been filled out correctly.

### Harms

All device-related adverse reactions (DAR) will be collected during intervention participants’ FES visits, or if a participant contacts their site FES clinician between visits. All serious adverse events (SAEs) will be reported to the Chief Iinvestigator and Peninsula Clinical Trials Unit (PenCTU) within 24 hours of the site becoming aware of the SAE.

A DAR is an adverse event considered to be caused by the trial intervention. An SAE or serious device-related adverse reaction:

- Results in death.
- Is life-threatening.
- Requires inpatient hospitalisation or prolongation of existing hospitalisation.
- Results in persistent or significant disability/incapacity.
- Is a significant or important medical event.

A suspected unexpected serious device-related adverse reaction is an event which is:

- Serious, as defined above.
- Considered to have been definitely, probably or possibly caused by either the trial intervention or the trial procedures.
- Deemed ‘unexpected’, that is, the reaction is one which has not been foreseen by the Chief Investigator.

SAEs will be detected by the research team at each of the data collection time points. Any events meeting the criteria for seriousness are subject to expedited reporting. For SAEs, the causal relationship between the SAE and trial participation will be assessed.

### Participant timeline

#### Screening

Potential participants will receive a pre-screening telephone call from the study team to determine whether they meet the eligibility criteria. This is determined through a combination of discussion and use of medical records. If they appear to be eligible and want to participate, they will be invited to a face-to-face screening visit.

During face-to-face screening, the researcher will receive informed consent, confirm eligibility and collect the following data:

- 10mWS.
- Gait characteristics.
- Demographics.
- Clinical history.
- Independence in activities of daily living.
- Walking ability.
- Sensory discrimination threshold (via 2-point discrimination and gradient discrimination tests).

#### Study visits

**Figure 2:**
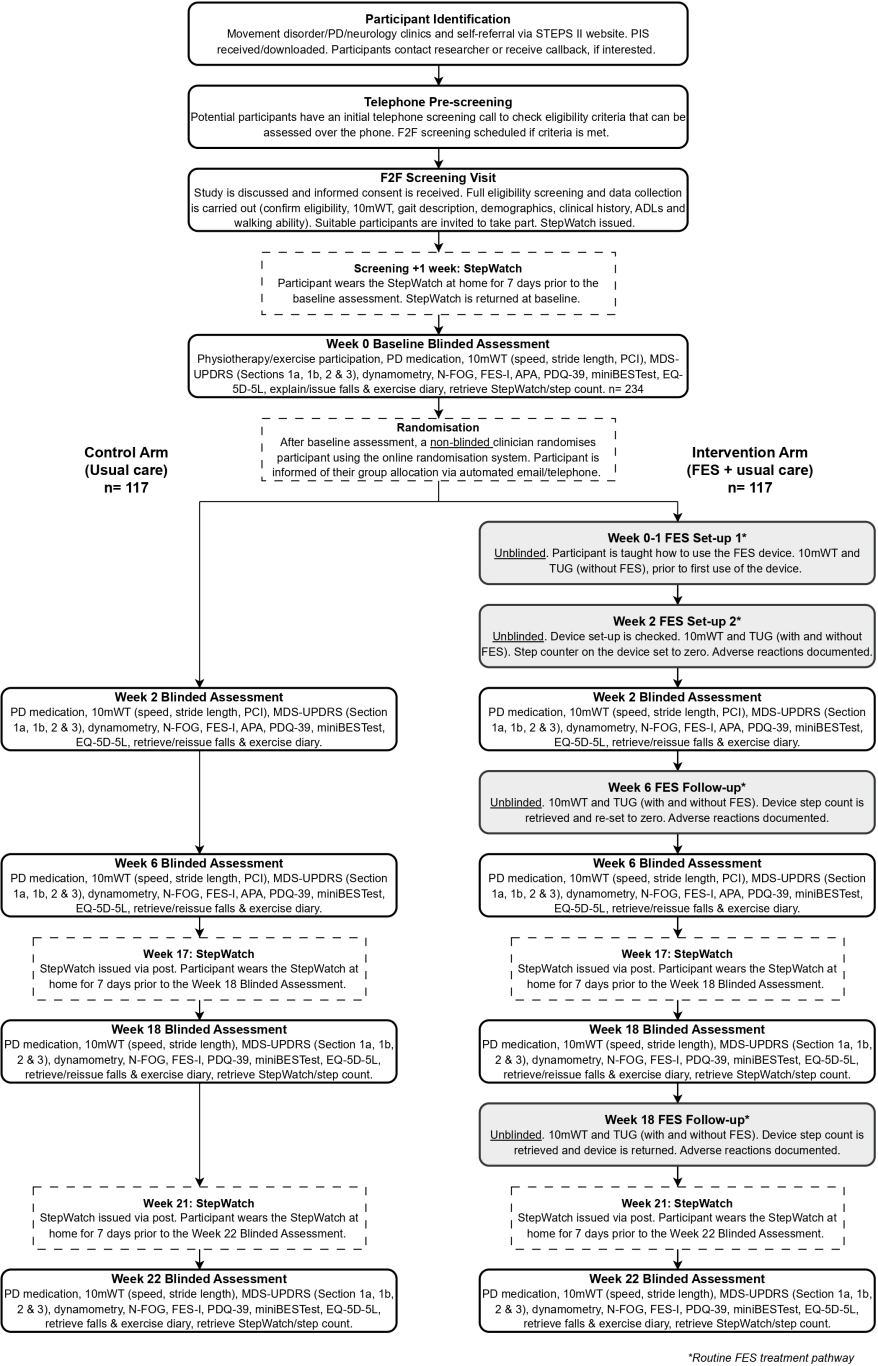
STEPS II randomised control trial participant pathway and study visits. 10mWT, 10 metre walking test; ADLs, activities of daily living; APA, anticipatory postural adjustment; EQ-5D-5L, European Quality of Life 5 Dimension 5 Level; F2F, face to face; FES, functional electrical stimulation; FES-I, Falls Efficacy Scale-International Questionnaire; MDS-UPDRS, Movement Disorder Society-Unified Parkinson’s Disease Rating Scale; miniBESTest, Mini-Balance Evaluation Systems Test; N-FOG, ‘New’ Freezing of Gait Questionnaire; PCI, Phase Coordination Index; PD, Parkinson’s disease; PDQ-39, Parkinson’s Disease Questionnaire-39; PIS, Participant Information Sheet; TUG, Timed Up and Go.

Figure 2 details the study visits. All participants have assessments at baseline (week 0) and weeks 2, 6, 18 and 22 with a blinded assessor.

Blinded assessments will be scheduled at a similar time each day to account for ‘on’ and ‘off’ medication periods and will take approximately 2 hours.

If the participant is in the intervention group, blinded assessments will take place on a different day to the unblinded FES visits.

Participants will also be issued with a Modus StepWatch pedometer and asked to wear it for 7 days prior to their baseline, week 18 and week 22 in-clinic assessments to monitor activity levels.

### Sample size

The sample size calculation is based on data from the randomised feasibility study. In total, 198 participants are required to detect a mean between-group difference in change in walking speed of 0.13 m/s at 18 weeks, assuming an SD of 0.28 m/s, with 90% power and two-sided significance level of 5%. To account for up to 15% of participants not reaching the primary endpoint, the target is to randomise 234 participants.

### Recruitment

Participants may be recruited at their routine neurology or physiotherapy outpatienet appointments. The study is also publicised via posters and leaflets displayed in clinical areas and social media accounts of participating NHS Trusts, PenCTU and Parkinson’s UK. Promotional materials signpost to the study website, where people can self-refer. After submission of the self-referral form, potential participants will receive a pre-screening call from the study team at their chosen site.

Recruitment performance is closely monitored by the Trial Management Group (TMG). Sites will maintain a screening log of potential participants, which is monitored to identify any barriers to recruitment.

### Randomisation

Participants will be individually randomised 1:1 to FES with usual care (intervention group) or usual care alone (control group) after their baseline assessment. Randomisation is stratified by a recruiting centre to ensure approximately equal numbers of participants in the intervention and control groups at each site. Randomisation is carried out via a secure, web-based randomisation system. The randomisation sequence, using variable block sizes, was generated by an independent statistician.

### Blinding

Participants or the treating clinician cannot be blinded to the allocation group due to the nature of the FES intervention. The individual carrying out assessments of outcome measures is blinded to group allocation. Intervention participants will be asked not to use FES on the assessment day or discuss their group allocation with the assessor. Blinding status will be checked at each assessment visit. At the week 22 assessment, the assessor will be asked to guess the group allocation. In the instance of unblinding, the assessor will be asked to provide a reason why unblinding occurred.

### Data management

PenCTU will oversee the management of data for the STEPS II trial. Participant screening and outcome data will be entered into REDCap Community, a web-based data capture system, by authorised personnel from participating NHS sites. Access to REDCap Community is regulated using usernames, encrypted passwords and two-factor authentication. Similarly, data concerning device tracking activities will be logged in a custom cloud-based system developed by PenCTU, accessible via authorised usernames and encrypted passwords. Both systems maintain electronic audit trails and validation mechanisms to ensure data integrity and security, including validation checks at the point of data entry.

### Statistical methods

A detailed statistical analysis plan (SAP) will be drafted by the trial statisticians and approved by an independent statistician prior to database lock.

Baseline characteristics of participants will be summarised descriptively and by group allocation and will be assessed for potential differences between participants who withdraw, discontinue FES and those who complete the trial. Loss to follow-up after randomisation will be reported separately for each group, summarised visually via a Consolidated Standards of Reporting Trials (CONSORT) flow diagram.

The analysis of the primary outcome measure will compare the change in walking speed between allocated groups using a normal linear mixed effects repeated measures model. The changes between baseline and each of the follow-up time points will be modelled on allocated group, time point and the interaction between allocated group and time point, with adjustment for baseline walking speed, recruitment site (stratification factor) and ankle proprioceptive sensory discrimination. Intercurrent events related to adherence to the intervention (ie, discontinuation/withdrawal) will be handled using the treatment policy strategy. A simple, unadjusted estimate of the between-group difference, and corresponding 95% CI, will also be calculated based on a two-sample t-test approach.

Prespecified sensitivity analyses will be undertaken to assess the robustness of the primary analysis results, specifically the missing at random assumption and carryover effect. Further sensitivity/secondary analyses of the primary outcome will be discussed and agreed with the TMG and oversight committees and prespecified in the SAP, including any exploratory subgroup analyses.

Analyses of the continuous secondary outcomes will follow the same approach as the main adjusted and unadjusted analyses of the primary outcome. Binary secondary outcomes, including the proportions of participants with an increase in walking speed of at least 0.13 m/s at week 18, will be analysed using logistic regression models, with adjustment for recruiting site.

Safety outcomes will be descriptively summarised on an as-treated basis; participants who attend at least one FES session will be categorised as being in the intervention group.

To address the postulated mechanistic hypotheses, a path analysis will be undertaken to assess the extent to which variability in foot movement (limb bradykinesia), strength, sensory threshold, interlimb coordination and APA at weeks 2 and 6 act as mechanisms for changing walking speed at weeks 6 and 18, respectively.

The trial includes an internal pilot phase. After the initial 12-month recruitment window, progress will be reviewed to inform whether the trial should progress to the main phase. Table 1 details the study progression criteria.

**Table 1:** Internal pilot: progression criteria after 12 months of recruitment

Progression criteria after 12 months of recruitment	Green band (progression of trial)	Amber band (progression of trial with remedial action)	Red band (likely termination of trial)
Sites open	7	4–6	<4
Participants recruited	≥100	71–99	<71
Retention rate (defined as completeness of primary outcome)	≥85%	75–84%	<75%

### Data Monitoring Committee

Unblinded accruing trial data and safety data will be reviewed at least annually by the independent Data Monitoring Committee, who will report their recommendations to the independent Trial Steering Committee, or sooner if there are participant safety concerns.

### Trial monitoring

To ensure data quality and completeness, the PenCTU data team will regularly monitor the data using validated R scripts. Details of these monitoring and validation activities are outlined in the data management plan, available on request. The monitoring process includes post-entry validation checks, ensuring completeness of critical data items, surveillance of safety and withdrawal reports, and centralised site monitoring. Central site monitoring may prompt on-site visits to verify data entry, consent procedures, eligibility criteria and other data-related aspects. The data manager will provide ongoing reports to the TMG regarding data quality and completeness throughout the trial.

Source data verification will be conducted at remote site monitoring sessions by PenCTU.

### Qualitative substudy

An embedded qualitative component will explore the experiences of pwPD and their carers/family members with FES. This data will contextualise the quantitative measures and help gain an understanding of how FES is used in the real world.

#### Aims

- Explore the views and experiences of pwPD using FES within the RCT.
- Explore the views and experiences of family members or carers of pwPD using FES within the RCT.

#### Design

An optional clause on the STEPS II trial consent form allows participants to opt in to the qualitative component. This involves 60 minute telephone or online semi-structured interviews with pwPD using FES and their family members or carers. There will be three participant groups:

- Group 1: a subsample of 30 pwPD randomised to the intervention arm; two interviews between weeks 14 and 18 and at week 22.
- Group 2: a subsample of pwPD randomised to the intervention arm who withdrew from using FES; one interview after they withdrew from the intervention.
- Group 3: a subsample of 15 family members or carers of individuals in group 1; one interview after their family member has stopped using FES.

#### Sampling and recruitment

A combination of convenience and purposive sampling approaches will be used to achieve a range of views based on diversity in age, gender, level of mobility, time since diagnosis of PD and recruitment centre using a sampling matrix. Purposive sampling will be used to recruit any gaps within the matrix. Those who are selected will be sent an invitation letter and Participant Information Sheet, with contact details for the qualitative researcher. If interested, participants will contact the researcher who will carry out telephone consent. After consent is received, a date and time for the interview will be scheduled.

#### Analysis

Data will be analysed using reflexive thematic analysis to identify key patterns relating to the experience of using FES via a combination of hand coding and NVivo V.11 software.

## Ethics and dissemination

### Research ethics approval

The study protocol and associated documents received approval from the Yorkshire and The Humber- Sheffield NHS Research Ethics Committee (REC) (reference 23/YH/0193). The study received funding from the National Institute for Health Research after independent external peer review. The study will be conducted in conformity with relevant regulations and the UK Policy Framework for Health and Social Care Research (2017).

### Dissemination policy

On completion of the trial, a final trial report will be prepared, submitted to the trial sponsor and funder and made publicly available.

The manuscript reporting the primary results will be submitted to a peer-reviewed medical journal as open access and reported in accordance with relevant CONSORT guidelines. A lay summary of trial results will be published on the PenCTU website and shared with participants. An anonymised participant-level dataset will be held within PenCTU.

In addition to a lay summary of the trial results, regular updates will be made available on the STEPS II website in the form of short updates and newsletters.

### Protocol amendments

Amendments requiring REC review will not be implemented until a favourable opinion is received. If required, amendments will be reviewed and accepted by the Health Research Authority (HRA) and NHS Research and Development departments before implementation at sites.

### Consent

Doctors, registered nurses or allied health professionals (band 5 or higher) may be authorised to receive informed consent for this study. Consent will only be provided after potential participants have had enough time to consider and discuss the study with their clinicians, family or friends. If they agree to take part, formal consent will be received by the principal investigator (PI) or delegated individual. The full inclusion and exclusion criteria will then be assessed.

The PI retains overall responsibility for the conduct of research at their site; this includes receiving informed consent from participants at their site. They must ensure that any person delegated the responsibility to participate in the informed consent process is duly authorised, trained and competent. If delegation of consent is acceptable, then details should be provided in the site delegation log. This will be monitored centrally by PenCTU.

### Confidentiality

To ensure participant confidentiality, the dataset will undergo anonymisation before any data dissemination.^48^ No direct identifiers will be shared with the trial statisticians, and access to directly identifiable information within the data capture system will be restricted. The trial adheres to relevant data protection legislation, including compliance with the HRA’s General Data Protection Regulation.^49^

### Ancillary and post-trial care

Participants in the intervention group return the FES device at the last unblinded visit. FES equipment is loaned to sites for the purpose of the study and must be returned to Salisbury NHS Trust after the study has concluded. Should participants wish to continue using FES after their final visit, they will be advised to speak to their FES clinician or general practitioner to explore any local options that may be available to them. Participants will be informed prior to agreeing to participate that the FES device is not currently part of the standard care for pwPD, and therefore may not be available to them on the NHS after their time on the study has concluded.

### Protocol and SAP

The protocol and SAP will be accessible via the study page on the ISRCTN register.
