# Supplementary material for: Efficacy of peroneal nerve functional electrical stimulation (FES) for the reduction of bradykinesia in Parkinson’s disease: an assessor-blinded randomised controlled trial (STEPS II)—study protocol
Source: BMJ Open. 2025 Sep 5;15(9):e097010. doi: 10.1136/bmjopen-2024-097010 (PMC12414162; doi:10.1136/bmjopen-2024-097010)
Supplement: Supplementary file 1 [file bmjopen-2024-097010.reviewer_comments.pdf]

PEER REVIEW HISTORY

BMJ Open publishes all reviews undertaken for accepted manuscripts. Reviewers are asked to complete a checklist review form and are provided with free text boxes to elaborate on their assessment. These free text comments are reproduced below.

ARTICLE DETAILS

Title (Provisional)

The Efficacy of Peroneal Nerve Functional Electrical Stimulation (FES) for the Reduction of Bradykinesia in Parkinson's Disease: An Assessor Blinded Randomised Controlled Trial (STEPS II): Study Protocol.

Authors

Tufft, Abbey; Neilens, Helen; Marsden, Jonathan; Creanor, Siobhan; Ali, Ayesha; Donovan-Hall, Margaret; Aspinall, Paigan; Lord, Amber; Jones, Ben; Taylor, Paul

---

VERSION 1 - REVIEW

**Reviewer**	**1**
**Name**	**Antoniades, Chrystalina**
**Affiliation**	**University of Oxford, Nuffield Department of Clinical Neurosciences**
**Date**	**10-Mar-2025**
**COI**	**None**

This is a well written and concise protocol in a much needed area. Abnormalities in walking in Parkinson's can diminish considerably quality of life in PwP and anything that can alleviate any issues should be supported. Well done to the authors for providing such a clear and thoughtful protocol.

The only minor comment I have for the authors is to consider how to relate their data to falls and near falls. There is mention in one of the questionnaires about falls but I would encourage the authors to consider how they potentially use some of the accelerometry data, see recent evidence for predicting falls in PD
https://www.nature.com/articles/s41746-024-01311-5

**Reviewer**	**2**
**Name**	**Scherbaum, Raphael**

**Affiliation**	**Ruhr University Bochum**
**Date**	**06-May-2025**
**COI**	**None**

This is a very well-written report of a study protocol for an ongoing and recruiting randomized controlled trial investigating the effects of 18-week daily peroneal nerve functional electrical stimulation on 10-meter walking speed in 234 people with Parkinson's disease compared to usual care. The trial was preceded by observational and smaller randomized controlled feasibility studies and is funded by a UK public agency after previous external peer-review. The manuscript provides a clear rationale for the study. The vast majority of information on methods is sufficiently detailed. Some minor details could be specified to enable possible replication, comply with the requirements of the BMJ Open submission guideline or SPIRIT checklist, or decrease minor ambiguities. I offer my comments as suggestions and hope you find them helpful. I was not able to comment on the quality of the design of the embedded qualitative study.

SPECIFIC COMMENTS

1. The page numbers given in the SPIRIT checklist should be adapted to correspond with the page numbers given in the manuscript. In addition, the 2025 update of the SPIRIT checklist has been published in the BMJ on 28 April 2025 by Chan et al. and should be considered to be applied.

2. My most important remark refers to factors possibly influencing the primary outcome, i.e., changes in mean 10-meter walking speed at week 18 compared to baseline. These factors include the amount of exercise and physiotherapy during usual care, the exact 'dose' or intensity of FES, and preconditions of motor learning like sufficient executive functions. Whether and how these factors are assessed could be made clearer. In detail, the ways of recording the 'data on usual care' (p11l31), the FES use (p11l21) and confounding factors like executive functions should be specified:

a. What data will be collected on usual care? Will the amount and intensity of exercise classes or physiotherapy be recorded systematically and included into the analyses? These factors may confound the primary outcome.

b. It could be made clearer that the intervention includes daily application of FES, especially in the abstract. Please clarify whether/how the actual 'dose' of FES each participant received will be determined. This can also be understood as reporting of a procedure for monitoring adherence as required by SPIRIT.

c. When relating to mechanisms of action, you could specify 'biomechanical' as an attribute, as 'mechanism of action' tends to be ambiguous. It lets me associate more neurophysiological measures and techniques such as fNRIS, fMRI, or EEG, that are not used by the study.

3. Comment on the mechanism of action: Adding to the immediate ‘orthotic effect’ of the stimulation, the ‘therapeutic effect' could be similar to effects observed with external somatosensory cues or tactile feedback. In fact, sensory electrical stimulation as a somatosensory cueing strategy for freezing of gait in PD has been investigated in three small observational studies (PMID: 30154990, PMID: 30154990, PMID: 30154990) and reviewed by Consentino et al. 2023 (PMID: 37086934). In PD, FES could be conceptualized as a simple closed-loop somatosensory cueing strategy. Therefore, mechanisms of action of FES may be in part similar to those proposed for cueing, involving goal-directed conscious control of gait or improved executive attention (PMID: 30907948, PMID: 38984716, PMID: 38858176, PMID: 35067999). Improvements in walking performance after FES may be associated with sufficient executive functions that are not captured by the study assessments. Therefore, it is possible that not all factors contributing to the FES effects are possible to be analysed (confounders of primary outcome). You could discuss this aspect at some point or in further work.

4. As requested by BMJ Open submission guidelines for study protocols, study dates such as overall study dates, registration dates and recruitment start/end dates should be added to clarify the status of data collection.

5. In the introduction, you may consider adding and paraphrasing your own seminal work (PMID: 22151049) two conference abstracts from other groups (https://doi.org/10.1016/j.clinph.2012.12.019,https://doi.org/10.1016/j.gaitpost.2024.08.019), and a review on FES highlighting the need for FES research in PD (PMID: 37259954).

6. Changes in eligibility criteria as outlined in the ISRCTN registry (https://doi.org/10.1186/ISRCTN13120555) could be mentioned, if applicable, i.e. if occurred during the ongoing data collection.

7. Inclusion criteria: Explain how the 1.25 ms-1 threshold for 10mWS was determined?

8. Did you determine any criteria of site eligibility?

9. Please comment on relevant concomitant care and interventions that are permitted or prohibited during the trial, e.g., physiotherapy.

10. The manufacturers and models of the IMU, pedometer, and dynamometer could be specified in more detail.

11. The APA stepping task could be described in more detail or provide a reference.

12. The trial outcome measures (ClinRO, PRO) could be complemented by references to their description, enabling appraisal of their reliability and validity. For example, the N-FOG has been reported to exhibit insufficient reliability and sensitivity to change which may limit the interpretation of this secondary outcome measure (PMID: 32071940).

13. While outcome measures for most objectives are detailed, methods to determine cost/utility are not described. These could be described briefly. For more clarity, you may additionally consider adding the corresponding domain from the objectives section to the outcome measures, e.g., stride length as an outcome measure of 'I. hypokinesia' or MDS-UPDRS II for 'IV ADL'.

14. Please indicate where the statistical analysis plan will be accessible.

15. Will the archived data stored in PEARL be publicly available individual de-identified participant data?

16. There is no declaration of competing interests from authors other than Paul Taylor. A statement on present or absent competing interests should be included.

VERSION 1 - AUTHOR RESPONSE

R1<br>C1	This is a well written and concise protocol in a much needed area. Abnormalities in walking in Parkinson's can diminish considerably quality of life in PwP and anything that can alleviate any issues should be supported. Well done to the authors for providing such a clear and thoughtful protocol.<br>The only minor comment I have for the authors is to consider how to relate their data to falls and near falls. There is mention in one of the questionnaires about falls but I would encourage the authors to consider how they potentially use some of the accelerometry data, see recent evidence for predicting falls in PD https://www.nature.com/articles/s41746-024-01311-5	Thank you for the positive feedback and this helpful suggestion. We agree that capturing information about falls is important. In the outcome measures section (starting on page 7), the final secondary outcome outlined is how weekly falls diaries are also being completed in addition to the fall's questionnaire (detailed in outcome measures section p8).<br>We are doing basic accelerometry outcome while doing the 10m walk test. This will give some information on balance and other gait parameters that could be related to the rate of falls.<br>Possible additional secondary analysis could be used to explore this further; the trial team will consider further this suggestion but given limited time/resource, such additional analyses may be beyond the scope of the trial.	
R2<br>C1	The page numbers given in the SPIRIT checklist should be adapted to correspond with the page numbers given in the manuscript. In addition, the 2025 update of the SPIRIT checklist has been published	Thank you for pointing this out. The Protocol paper has been added to the SPIRIT template in response to the editor comment, the separate SPIRIT checklist is no longer required.	

	in the BMJ on 28 April 2025 by Chan et al. and should be considered to be applied.		
R2<br>C2	My most important remark refers to factors possibly influencing the primary outcome, i.e., changes in mean 10-meter walking speed at week 18 compared to baseline. These factors include the amount of exercise and physiotherapy during usual care, the exact 'dose' or intensity of FES, and preconditions of motor learning like sufficient executive functions. Whether and how these factors are assessed could be made clearer. In detail, the ways of recording the 'data on usual care' (p11l31), the FES use (p11l21) and confounding factors like executive functions should be specified:<br><br>a. What data will be collected on usual care? Will the amount and intensity of exercise classes or physiotherapy be recorded systematically and included into the analyses? These factors may confound the primary outcome.<br><br>b. It could be made clearer that the intervention includes daily application of FES, especially in the abstract. Please clarify whether/how the actual 'dose' of FES each participant received will be determined. This can also be understood as reporting of a procedure for monitoring adherence as required by SPIRIT.<br><br>c. When relating to mechanisms of action, you could specify 'biomechanical' as an attribute, as 'mechanism of action' tends to be ambiguous. It lets me associate	Thank you for outlining these points of consideration. In relation to a) PD medication, frequency/type exercise, physiotherapy attendance, we have added clarity in the Intervention and Comparator section: "Participants in the control group will receive their usual care. Usual care includes PD medication (dose/frequency) and participation in physiotherapy or exercise (type of exercise/duration). Data on usual care will be collected for all participants; medication will be recorded at each blinded assessment and participation in exercise and physiotherapy will be recorded by the participant in weekly exercise diaries."<br><br>b) Added 'daily FES use' to abstract. Added detail to "Intervention and Comparator" section about dose: "At each FES appointment, the clinician will extract data from the ODFS Pace logger on number of steps taken whilst using the device, total stimulation time, intensity and pulse width to determine frequency of device use and adherence in between visits."<br><br>c) Thank you for raising this point, which may relate to us not explaining in the introduction about how we are investigating the possible mechanism or if we have an hypothesis that we are testing. In the grant application we say "Mechanisms of action will be explored by assessing short-term changes in inter-limb coordination, dynamic balance and limb bradykinesia and assessing their relative contributions in improving walking speed after 6 and 18 weeks. Changes in weakness in muscles directly targeted and not targeted by the intervention will be characterised over time. Neurophysiological may not be enough to fully explain this." The above wording from the grant application has been added to the background for clarity.	a) 7<br>b) 1, 7<br>c) 4

	more neurophysiological measures and techniques such as fNRIS, fMRI, or EEG, that are not used by the study.		
R2<br>C3	Comment on the mechanism of action: Adding to the immediate 'orthotic effect' of the stimulation, the 'therapeutic effect' could be similar to effects observed with external somatosensory cues or tactile feedback. In fact, sensory electrical stimulation as a somatosensory cueing strategy for freezing of gait in PD has been investigated in three small observational studies (PMID: 30154990, PMID: 30154990, PMID: 30154990) and reviewed by Consentino et al. 2023 (PMID: 37086934). In PD, FES could be conceptualized as a simple closed-loop somatosensory cueing strategy. Therefore, mechanisms of action of FES may be in part similar to those proposed for cueing, involving goal-directed conscious control of gait or improved executive attention (PMID: 30907948, PMID: 38984716, PMID: 38858176, PMID: 35067999). Improvements in walking performance after FES may be associated with sufficient executive functions that are not captured by the study assessments. Therefore, it is possible that not all factors contributing to the FES effects are possible to be analysed (confounders of primary outcome). You could discuss this aspect at some point or in further work.	In the feasibility study, we looked at the improvements in function from cueing interventions and saw that the effect seen in the feasibility work was significantly greater than control, particularly in carryover. We therefore concluded that it was likely that other mechanisms were involved. Therefore, in this definitive trial, we are assessing the 10mwt at follow-up with and without FES in participants in the intervention group and so this could show a direct cueing effect. The pilot data showed the effect was less than the training effect.	
R2<br>C4	As requested by BMJ Open submission guidelines for study protocols, study dates such as overall study dates, registration dates and recruitment start/end	Added a 'Study Dates' section; please advise if this should be removed (there is not a section in the BMJ Open spirit template to add study dates).	16

	dates should be added to clarify the status of data collection.		
R2 C5	In the introduction, you may consider adding and paraphrasing your own seminal work (PMID: 22151049) two conference abstracts from other groups (https://doi.org/10.1016/j.clinph.2012.12.019,https://doi.org/10.1016/j.gaitpost.2024.08.019), and a review on FES highlighting the need for FES research in PD (PMID: 37259954).	Pilot and feasibility work is reference in the introduction (ref 31-33). Added conference abstracts and FES review to references in the introduction.	4
R2 C6	Changes in eligibility criteria as outlined in the ISRCTN registry (https://doi.org/10.1186/ISRCTN13120555) could be mentioned, if applicable, i.e. if occurred during the ongoing data collection.	Eligibility criteria were updated prior to commencement of recruitment (updated Oct 23, Recruitment commenced Jan 24).	
R2 C7	Inclusion criteria: Explain how the 1.25 ms-1 threshold for 10mWS was determined?	Following detail added to Eligibility Criteria section:<br>"The maximum 10m walking speed for inclusion in the study was raised from 0.8 to 1.25ms-1, five months into the feasibility study. This was because many pwPD who were initially screened for the study were rejected at assessment, despite reduced ankle movements, because they walked too fast. Increasing the threshold for inclusion to 1.25ms-1 better reflects the cohort of pwPD who have walking problems. This speed also has practical meaning for community mobility as it is the minimum speed needed to safely use a pelican crossing"	6
R2 C8	Did you determine any criteria of site eligibility?	Added further detail to 'Trial design and setting' section: "Participating sites were assessed for feasibility by presence of an established FES service and suitable staff to assume the blinded assessor role who had experience assessing walking in pwPD."	5
R2 C9	Please comment on relevant concomitant care and interventions that are permitted or prohibited during the trial, e.g., physiotherapy.	Our response to, and clarifications in the revised paper, R3a response cover this point - usual care is permitted, including physiotherapy. Exclusion criteria detail the medications which are not permitted (DBS), scheduled to start apomorphine or (pro)dodopa).	
R2 C10	The manufacturers and models of the IMU, pedometer, and dynamometer could be specified in more detail.	Specified manufacturer in the 'Outcomes' section: Physilog (IMU), Modus (StepWatch), Activforce (dynamometer).	8
R2 C11	The APA stepping task could be described in more detail or provide a reference.	Reference added	8

R2<br>C12	The trial outcome measures (ClinRO, PRO) could be complemented by references to their description, enabling appraisal of their reliability and validity. For example, the N-FOG has been reported to exhibit insufficient reliability and sensitivity to change which may limit the interpretation of this secondary outcome measure (PMID: 32071940).	Added references for outcome measures.	8
R2<br>C13	While outcome measures for most objectives are detailed, methods to determine cost/utility are not described. These could be described briefly. For more clarity, you may additionally consider adding the corresponding domain from the objectives section to the outcome measures, e.g., stride length as an outcome measure of 'I. hypokinesia' or MDS-UPDRS II for 'IV ADL'.	Added "The clinician will record consumables used and contact time; this data will be used to determine cost/utility of intervention delivery."	7
R2<br>C14	Please indicate where the statistical analysis plan will be accessible	New heading added "Protocol and statistical analysis plan" stating "The protocol and SAP will be accessible via the study page on the ISRCTN register."	15
R2<br>C15	Will the archived data stored in PEARL be publicly available individual de-identified participant data?	Yes – this data will be open access and de-identified.	
R16	There is no declaration of competing interests from authors other than Paul Taylor. A statement on present or absent competing interests should be included.	Added "There are no competing interests to declare from other authors."	16
